# Fully Roll-to-Roll Processed Efficient Perovskite Solar Cells via Precise Control on the Morphology of PbI_2_:CsI Layer

**DOI:** 10.1007/s40820-022-00815-7

**Published:** 2022-03-25

**Authors:** Hengyue Li, Chuantian Zuo, Dechan Angmo, Hasitha Weerasinghe, Mei Gao, Junliang Yang

**Affiliations:** 1 Hunan Key Laboratory of Nanophotonics and Devices, School of Physics and Electronics, Central South University, Changsha, 410083 People’s Republic of China; 2 Flexible Electronics Laboratory, CSIRO Manufacturing, Clayton, VIC 3168 Australia

**Keywords:** Perovskite solar cells, Slot-die coating, Roll-to-roll, Ambient condition, Flexible

## Abstract

Perovskite solar cells (PSCs) have attracted tremendous attention as a promising alternative candidate for clean energy generation. Many attempts have been made with various deposition techniques to scale-up manufacturing. Slot-die coating is a robust and facile deposition technique that can be applied in large-area roll-to-roll (R2R) fabrication of thin film solar cells with the advantages of high material utilization, low cost and high throughput. Herein, we demonstrate the encouraging result of PSCs prepared by slot-die coating under ambient environment using a two-step sequential process whereby PbI_2_:CsI is slot-die coated first followed by a subsequent slot-die coating of organic cations containing solution. A porous PbI_2_:CsI film can promote the rapid and complete transformation into perovskite film. The crystallinity and morphology of perovskite films are significantly improved by optimizing nitrogen blowing and controlling substrate temperature. A power conversion efficiency (PCE) of 18.13% is achieved, which is promising for PSCs fabricated by two-step fully slot-die-coated devices. Furthermore, PSCs with a 1 cm^2^ area yield a champion PCE of 15.10%. Moreover, a PCE of 13.00% is obtained on a flexible substrate by the roll-to-roll (R2R) coating, which is one of the highest reported cells with all layers except for metal electrode fabricated by R2R process under ambient condition.

**Supplementary Information:**

The online version contains supplementary material available at 10.1007/s40820-022-00815-7.

## Highlights

- The slot-die-coated porous PbI_2_:CsI film assisted with nitrogen blowing can promote the rapid and complete transformation of perovskite film.
- The crystallinity and morphology of slot-die-coated perovskite film are significantly improved by controlling substrate temperature.
- Fully slot-die-coated perovskite solar cells achieve a power conversion efficiency (PCE) of 18.13%, and fully roll-to-roll printed flexible PSCs achieve a PCE of 13.00% in ambient condition.

**Supplementary Information:**

The online version contains supplementary material available at 10.1007/s40820-022-00815-7.

## Graphical abstract

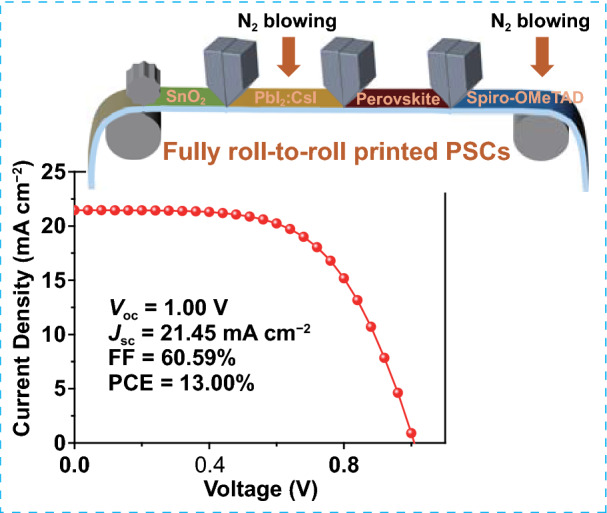

## Introduction

The organic–inorganic hybrid perovskite solar cells (PSCs) are considered one of the promising new-generation solar cells. The certified power conversion efficiency (PCE) has reached a remarkable value of 25.7% since the first report in 2009 [1, 2]. These achievements can be attributed to the intrinsic properties of the perovskite material, such as a large absorption coefficient, high charge mobility, low exciton binding energy and low temperature solution processing ability [3–8]. Numerous studies have been attempted to achieve high-quality perovskite films since then, including additives [9–12], solvent engineering [13–15], interface modification [16, 17], and scale-up techniques under ambient fabrication environment [18–22], which would considerably move the development of PSCs to commercialization.

However, most high-performance PSCs are mainly fabricated via spin-coating method due to their accessibility, high repeatability, facile control and suitability for anti-solvent [23, 24]. The major drawback is that spin-coating is unsuitable and difficult to match other scalable printing processes. Recently, growing investigations on PSCs prepared by scalable methods such as blade-coating and slot-die coating have been demonstrated, which are transferable and compatible with the roll-to-roll (R2R) process [25–27]. Among various scalable coating techniques, slot-die coating stands out owing to its high reported PCE, fast coating speeds, high material utilization, and matching with the R2R process [28–30]. It is well known that perovskite film plays an essential role in the performance of PSCs. Both one-step and two-step deposition methods have been broadly employed in the fabrication of PSCs. In one-step perovskite deposition, it is difficult to modulate the crystal growth for forming a uniform film, especially on a large scale [31]. While in the two-step deposition, PbI_2_ layer is firstly coated on the substrate by various methods and usually show excellent coverage, which is considered more suitable and reliable for the mass production process. Kim et al. demonstrated a two-step deposition of PSCs with a PCE of 10.9% by fully gravure printing and a PCE of 9.7% by partly R2R process [32]. Later, they adopt tert-butyl alcohol as an anti-solvent to obtain a wide processing window and achieved a PCE of 13.8% for fully R2R processed PSCs [33]. Burkitt et al. used fully R2R process to print p-i-n PSCs with a PCE of 12.2% [34]. Recently, Othman et al. demonstrated that fully R2R slot-die-coated triple-cation PSCs in ambient condition with underlying guanidinium iodide in hole transport layer showed a PCE of 12% [35]. Following the pioneering work by Gratzel group [36], high-performance and stable triple-cation planar heterojunction (PHJ) PSCs have been fabricated successfully via a low temperature sequential solution process [37, 38]. The device with ITO/SnO_2_/Perovskite/Spiro-OMeTAD/Ag exhibited a PCE of over 20% by spin coating. Meanwhile, devices can sustain about 80% of the initial PCE when stored in air (humidity = 40%) for over 500 h without any encapsulation. Therefore, if the PSCs fabricated by the scalable method can achieve similar PCEs, especially by R2R coating process, it would tremendously accelerate the commercialization process of PSCs.

In this work, we demonstrate the fabrication of fully slot-die-coated PSCs with a n-i-p structure in ambient condition using a two-step process. All layers of PSC devices were prepared by scalable slot-die coating process except the evaporated metal electrode. The initially formed PbI_2_:CsI film showing porous morphology is able to facilitate the fast and complete conversion of PbI_2_:CsI film to a pin-hole-free perovskite film which was assisted with heating and N_2_ blowing, leading to the fully slot-die-coated PSC devices with a max PCE of 18.13%. Furthermore, devices with a 1 cm^2^ area yielded a champion PCE of 15.10%. Remarkably, R2R processed PSC devices achieved a maximum PCE of 13.00%. The results provide significant and continued inspiration for processing high-performance, large-area flexible PSCs, which is helpful for promoting the potential commercialization of PSCs.

## Experimental Details

### Materials

All of the chemical materials were used directly without any purification, including tin oxide precursor (SnO_2_, 15% in H_2_O colloidal dispersion, Alfa Aesar), lead iodide (PbI_2_, 99%, Greatcell Solar), cesium iodide (CsI, 99%, Strem Chemicals, inc.), formamidinium iodide (HC(NH_2_)_2_I, 99.5%, Greatcell Solar), methylammonium chlorine (CH_3_NH_3_Cl, 99.5%, Xi'an Polymer Light Technology Corp.), methylammonium bromide (CH_3_NH_3_Br, 99.5%, Greatcell Solar), 2,20,7,70-tetrakis-(N,N-di-4-methoxyphenylamino)-9,90-spirobifluorene (Spiro-OMeTAD, 99%, Xi'an Polymer Light Technology Corp.), lithium bis(trifluoromethanesulfonyl)imide (Li-TFSI, 97%, Sigma-Aldrich), 4-tert-butylpyridine (4-tBP, 98%, Sigma-Aldrich), isopropanol (IPA, 99.5%,Sigma-Aldrich), N,N-dimethylformamide (99.8%, Sigma-Aldrich), chlorobenzene (99.8%, Sigma-Aldrich), and acetonitrile (ACN, 99.95%, Sigma-Aldrich). ITO glass substrates were purchased from Shenzhen Display, China.

### Materials Characterization

UV-Vis spectra were recorded on a Lambda 35 Perkin-Elmer absorption spectrometer. PL spectra were recorded using a fluorescence spectrophotometer (LS55, Perkin-Elmer). XRD patterns were obtained using a Bruker D8 Advance X-ray Diffractometer operating under Cu Kα radiation (40 kV, 40 mA) equipped with a LynxEye detector. The SEM images of the films were taken with a Zeiss Merlin field emission SEM.

### Device Fabrication

#### Slot-Die Coated Devices on ITO/glass

The ITO/glass substrate was ultrasonically cleaned using detergents/H_2_O, distilled water, acetone and isopropanol for 5 min sequentially. Then, dried by clean N_2_ flow and treated by UV-ozone for 15 min at room temperature. For the electron transport layer, the SnO_2_ nanoparticles solution that was diluted by H_2_O to half of the original concentration and filtered using a 0.22 μm PVDF filter, was deposited by slot-die coating at a speed of 5 mm s^−1^ with a 1 µL cm^−2^ solution in ambient air, and followed by a post-annealing at 150 °C for 30 min. Because SnO_2_ layer has been extensively used for the slot-die coating for PSCs, and an optimal thickness for perovskite devices has also been obtained [39], thus no further optimization was conducted in this work. After depositing the SnO_2_ film, the perovskite layer was formed via a two-step slot-die coating deposition in ambient condition. The mixture solution of PbI_2_:CsI (599.3 mg: 33.8 mg in 900 µL DMF and 100 µL DMSO) was slot-die coated at a head moving speed of 5 mm s^−1^ with a 1 µL cm^−2^ solution feed rate on the stationary SnO_2_ layer, and a gap between the slot-die head and the substrate was controlled at 200 µm. The slot-die coated PbI_2_:CsI films were heated at 70 °C for 3 min in ambient air. After cooling down of the PbI_2_:CsI film, the mixture solution of FAI: MABr: MACl in IPA was slot-die coated at a speed of 2 mm s^−1^ with a 1 µL cm^−2^ solution feed on the PbI_2_:CsI film without heating, and then post-annealed at 150 °C for 15 min in air. Subsequently, the Spiro-OMeTAD solution was drop casted or slot-die coated assisted by heating and gas blowing in ambient condition, where 1 mL Spiro-OMeTAD/chlorobenzene (90 mg mL^−1^) solution was employed with the addition of 10 µL tBP and 45 µl Li-TFSI/ACN (170 mg mL^−1^). Finally, 100 nm-thick Ag electrode was deposited through thermal evaporation with a mask at a pressure of 8 × 10^–6^ mbar, resulting in an active area of 0.1 or 1 cm^2^.

#### R2R Coated Devices on Flexible ITO/PET

The R2R coating of SnO_2_ was carried out by the reverse-gravure coating method, using a Mino-Labo™printer (MAHY-1310; Yasui Seiki Co.Ltd). The coating was conducted at 0.16 m min^−1^ bed speed and 16 rpm of 13 mm wide reverse-gravure roll speed to deposit the 13 mm wide continuous SnO_2_ layer in the middle of the ITO/PET substrate (width: 25 mm). The coated wet SnO_2_ film was dried on-line at 135 °C for 10–12 seconds on a hot plate to remove the solvent. A PbI_2_:CsI solution (599.3 mg: 33.8 mg in 900 μL DMF and 100 μL DMSO) was slot-die coated on top of the SnO_2_ surface (70 °C) at the feed rate of 18 μL min^−1^ and the bed speed of 0.3 m min^−1^. The formed PbI_2_:CsI film was then annealed at 70 °C assisted with N_2_ blowing. The perovskite conversion was conducted by slot-die coating of a mixed solution of FAI: MABr: MACl (60 mg: 6 mg: 6 mg in 1 mL IPA) at a bed speed of 0.3 m min^−1^, solution feed rate of 40 μL min^−1^ followed by annealing at 135 °C. Finally, Spiro-OMeTAD was slot-die coated at the bed temperature of 60 °C, bed speed of 0.3 m min^−1^ and feed rate of 40 uL min^−1^ without further annealing. The completed film was cut into 2.5 cm × 2.5 cm and 100 nm-thick Ag electrode was deposited through thermal evaporation as described in Sect. 2.3.1.

### Device Measurements

Current density–voltage (*J-V*) curves of the devices were measured by a Keithley 2400 Source Meter under standard solar irradiation (AM 1.5 G, 100 mW cm^−2^). The light intensity was calibrated using a reference cell (Hamamatsu S1133 with KG5 filter and 2.8 × 2.4 mm^2^ of photosensitive area), which was calibrated by a certified reference cell (PV Measurements, certified by NREL) under 1000 W m^−2^ AM 1.5G illumination from a Newport LED lamp source with a ABA grade spectrum.

## Results and Discussion

### Fabrication of Perovskite Film

**Fig. 1:**
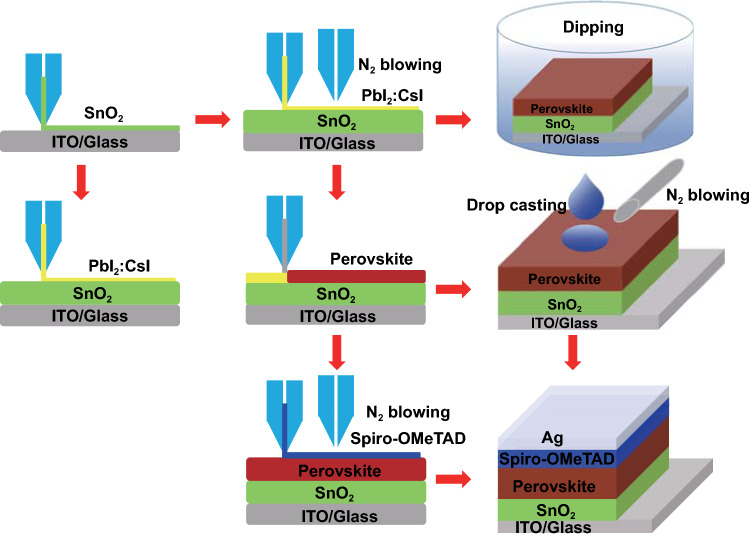
Schematic of the device fabrication process

Figure 1 shows the fabrication schematic of PSCs using the two-step process with a device structure of ITO glass/SnO_2_/Perovskite/Spiro-OMeTAD/Ag. Briefly, SnO_2_ was slot-die coated on glass, followed by PbI_2_:CsI film. The quality of the PbI_2_:CsI film was first checked by a dipping process into the cations containing solution. Thereafter, the cation containing was also slot-die coated followed by drop casting of the hole transport layer. Finally, the hole transport layer of Spiro-OMeTAD was also slot-die coated, resulting in fully slot-die coated devices.

**Fig. 2:**
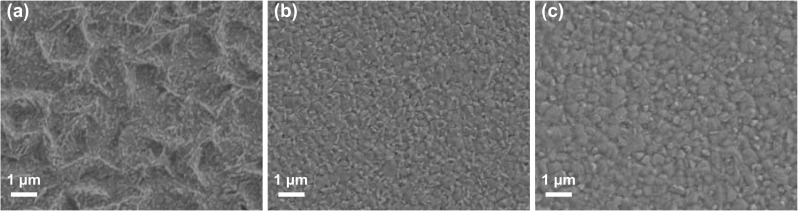
**a**, **b** SEM images of perovskite films dipped in the 30 and 60 mg mL^−1^ FAI/MABr/MACl solution, respectively. **c** SEM image of perovskite film formed from slot-die coated FAI/MABr/MACl solution at 60 mg mL^−1^

The quality and density of the PbI_2_:CsI film strongly affect the perovskite film quality in a two-step process. Heating is a common way to dry film to reduce the effect of humidity when the wet film is deposited [40–42]. The PbI_2_:CsI film tends to form a dense layer if drying dynamics is not controlled, as shown in Fig. S1a. Hence, an additional slot-die head was attached to channel N_2_ blowing over the freshly slot-die coated PbI_2_:CsI wet film as shown in Fig. 1. N_2_ blowing enables homogenous and porous morphology formation of the PbI_2_:CsI film, which provides channels facilitating cations to penetrate throughout the bulk of the PbI_2_:CsI film and enable formation of a pin-hole free and fully converted homogenous perovskite film. PSCs have been demonstrated to be more efficient and stable by combining MA, FA and Cs cations [43–45]. Therefore, a similar strategy herein was employed to fabricate perovskite films. For comparison, the PbI_2_:CsI films were dipped into the FAI/MABr/MACl solution at two different concentrations of 30 and 60 mg mL^−1^, respectively. The scanning electron microscope (SEM) images of the resulting films are shown in Fig. 2a, b. Clearly, the formed perovskite film at the higher concentration displays a much smoother surface than that one with the lower concentration. Hence, a 60 mg mL^−1^ FAI/MABr/MACl solution was used for further optimization and translation to slot-die coating, unless otherwise specified. One of the striking advantages of using slot-die coating compared to dipping is that much less perovskite precursor solution is required for each device. Accordingly, slot-die coating was adopted for the deposition of FAI/MABr/MACl solution. As expected, a high-quality perovskite film was achieved through this sequential process (Fig. 2c).

**Fig. 3:**
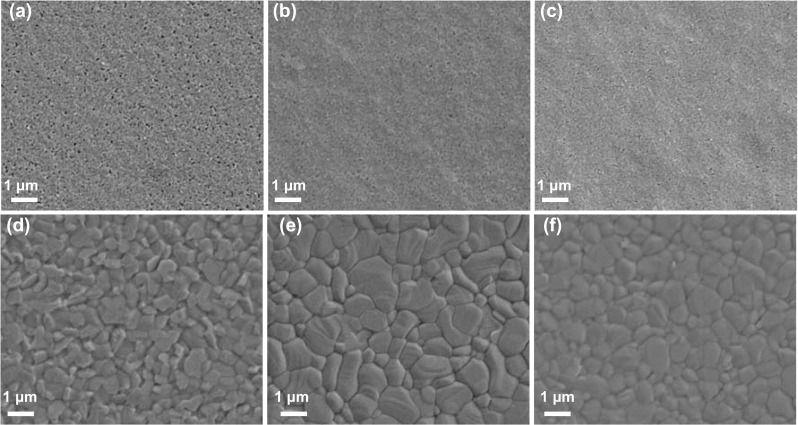
**a–c** SEM images of slot-die coated PbI_2_:CsI films under ambient conditions at the different bed temperatures of 60, 70, and 80 °C, respectively. **d–f** SEM images of slot-die coated perovskite films under ambient conditions at the different bed temperatures of 60, 70, and 80 °C

In the two-step fabrication process, the quality and density of PbI_2_:CsI films are significant keys to facilitating the efficient conversion, leading to the desired perovskite films. The gas blowing and substrate-heating approaches are important factors that can be utilized in tandem with slot-die coating of solution to achieve desired films. Both of them can also minimize the influence of surrounding humidity to improve perovskite crystallization when being processed under an ambient environment [46]. The SnO_2_-coated substrates were heated at the different bed temperatures, and the PbI_2_:CsI films were deposited on top of the heated substrates. The morphologies of PbI_2_:CsI films are well controlled by precise adjustment of the bed temperature and monitored by SEM. As shown in Fig. 3a-c, there are more pin-holes present within the PbI_2_:CsI film when prepared at 60 °C than that at 70 °C. When the bed temperature was further increased to 80 °C, the PbI_2_:CsI films tend to become compact, which is attributed to the fact that the solvent evaporates much faster at the higher temperatures, causing PbI_2_:CsI crystals to precipitate quickly, forming a densely packed film. Figures 3d-f are the SEM images of perovskite films formed on the above-deposited PbI_2_:CsI films at room temperature via slot-die coating of FAI/MABr/MACl solution. The perovskite grain size is summarized by analyzing the size of 150 grains through Nano Measurer software, as shown in Fig. S2. The grain size increases initially along with the bed temperature changing from 60 to 70 °C, and then decreases when the bed temperature reached 80 °C. This trend can be attributed to the supersaturation of PbI_2_ crystals at high bed temperature, leading to the excessive incomplete converted PbI_2_, which will be discussed in detail below. The optimal number of pores would provide enough space for FAI/MABr/MACl to diffuse into PbI_2_:CsI films, enabling complete conversion to perovskite. On the contrary, too densely packed PbI_2_:CsI film hinders the solution diffusion into the PbI_2_:CsI bulk film, inhibiting efficient interaction between PbI_2_:CsI and FAI/MABr/MACl, causing incomplete conversion to perovskite and negatively impacting device performance. Therefore, the quality of PbI_2_:CsI films controlled by bed temperature exhibits considerable effects on the fabrication of high-quality perovskite films. Thus, the results suggest that an optimal porous morphology of the PbI_2_:CsI film is critical to form smooth, homogeneous, fully converted perovskite films with large grains, while a dense PbI_2_:CsI layer leads to the incomplete conversion into perovskite [47].

### Characterization of Perovskite Film

**Fig. 4:**
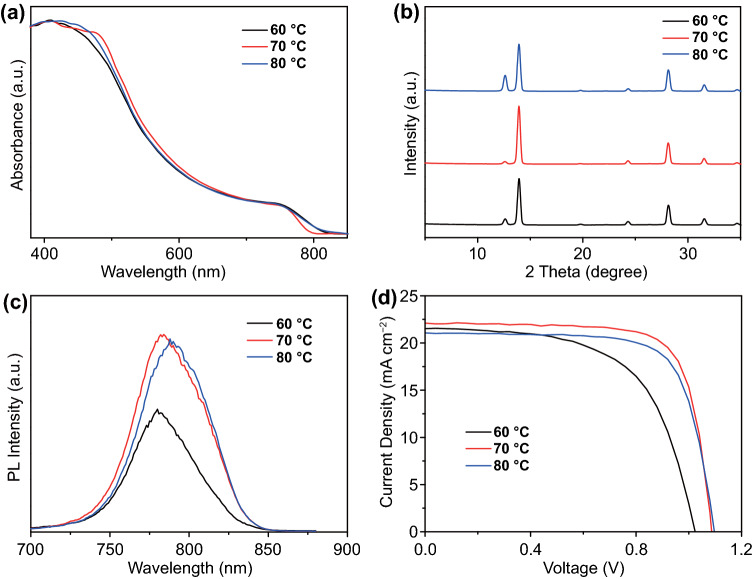
**a** Absorbance spectra, **b** XRD patterns, **c** photoluminescence and **d** *J–V* curves of perovskite films prepared using the PbI_2_:CsI films fabricated on the different bed temperatures

Figure 4a shows UV–vis absorption spectra of the perovskite films fabricated at different bed temperatures using a standard detector. All the perovskite films exhibit similar absorption as previously reported. The XRD results are displayed in Fig. 4b. When the bed temperature increased to 80 °C, a small peak at 2*θ* = 12.7° distinctly appeared in the XRD patterns. This typical peak is ascribed to the unreacted excessive PbI_2_ crystals that primarily reside at the bottom of perovskite film. For investigating the effect of bed temperature on photogenerated charge carriers of the perovskite film, the steady-state photoluminescence (PL) spectra were measured on perovskite films prepared on glass substrate using the same two-step process (Fig. 4c). The perovskite film fabricated at 70 °C presents the highest PL intensity, implying that the nonradiative recombination of perovskite film was significantly suppressed at this bed temperature. The PL peak of perovskite film fabricated at 80 °C has a small redshift, which indicates a higher trap density in perovskite film [48]. Therefore, the increased recombination loss and reduced charge collection of the perovskite film prepared at 80 °C will have a negative influence on the solar cell performance.

The *J-V* curves and performance parameters of PSC devices fabricated from the above perovskite films prepared at different bed temperatures are shown and summarized in Fig. 4d and Table 1, respectively. Slot-die coating was used for the deposition of SnO_2_ and perovskite layers, except that Spiro-OMeTAD hole transport layer was coated by drop-casting. The short-circuit current (*J*_*sc*_) increased when the bed temperature increased from 60 to 70 °C. On the contrary, the *J*_*sc*_ slightly decreased with increasing the bed temperature to 80 °C. The highest *J*_*sc*_ of 22.11 mA cm^−2^ was achieved at the optimized bed temperature of 70 °C. Meanwhile, the fill factor (*FF*) was also improved to 75.2%, resulting in the final PCE of 17.96%. The achieved device results comply with the previous result analysis from SEM, PL and XRD. Conspicuously, a suitable bed temperature is crucial in determining PbI_2_:CsI film morphology and in turn final device performance. The bed temperature at 80 °C accelerates solvent evaporation, resulting in the unavoidable high density of PbI_2_:CsI film, which causes the incomplete conversion of PbI_2_:CsI to perovskite. At this point, slot-die coating as an effective way to deposit PbI_2_:CsI and perovskite film at the certain temperatures range has been demonstrated. This setup is believed to be readily transferred to the R2R continuous process.

**Table 1:** Average values of photovoltaic parameters obtained from *J–V* measurements for PSCs derived from PbI_2_:CsI prepared by slot-die coating under ambient conditions at the different bed temperature

Temperature (°C)	*V*_oc_ (V)	*J*_sc_ (mA cm^−2^)	*FF* (%)	*PCE* (%)	*PCE* (Max)
60	1.05 ± 0.04	20.90 ± 0.72	58.42 ± 3.39	12.90 ± 0.94	13.98
70	1.08 ± 0.02	22.09 ± 0.35	74.30 ± 1.12	17.80 ± 0.13	17.96
80	1.07 ± 0.02	21.29 ± 0.32	71.22 ± 2.38	16.28 ± 0.55	16.86

The performance parameters are the average values of 8 devices

### Photovoltaic Performance of Fully Slot-Die Coated PSCs

**Fig. 5:**
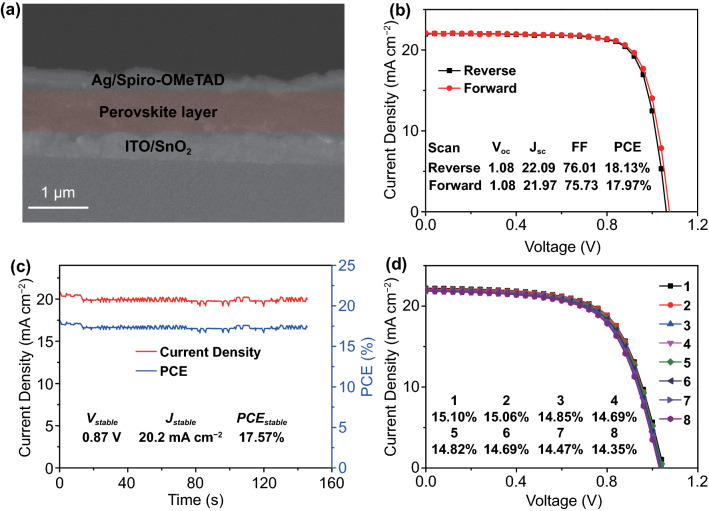
**a** SEM cross-sectional image for a fully slot-die coated PSC except for the electrode. **b** *J–V* curves of the champion cell under reverse and forward scan. **c** Steady-state PCE of the champion cell. **d** *J–V* curves of 8 PSCs with 1 cm^2^ area fabricated by fully slot-die coating except for the electrode

Notwithstanding the impressive performance parameters were obtained from the slot-die coated perovskite layer on the glass substrate, the industrial compatible process requires all layers to be fabricated with scalable deposition strategies. Figure 5a shows a SEM cross-section of the PSCs, in which all layer but the electrodes, namely SnO_2_, perovskite layer and Spiro-OMeTAD layer, were slot-die coated. The thickness of the perovskite film reaches about 650 nm. The *J-V* curves of the best performance of PSCs are shown in Fig. 5b. The champion cell exhibits an open-circuit voltage (*V*_*oc*_) of 1.08 V, a *J*_*sc*_ of 22.09 mA cm^−2^, an *FF* of 76.01%, and a PCE of 18.13% in a reverse scan. A steady-state PCE of 17.57% was obtained (Fig. 5c). Extending slot-die coating to large-area fabrication, 1 cm^2^ device was fabricated via the same process (Fig. S3). *J-V* curves of 8 PSC devices are presented in Fig. 5d, and the corresponding PCEs are shown in the inset. The champion 1 cm^2^ device shows a PCE of 15.10%, which demonstrates that large-area PSCs could be effectively fabricated by slot-die coating in ambient conditions.

### Fully R2R Processed PSCs

**Fig. 6:**
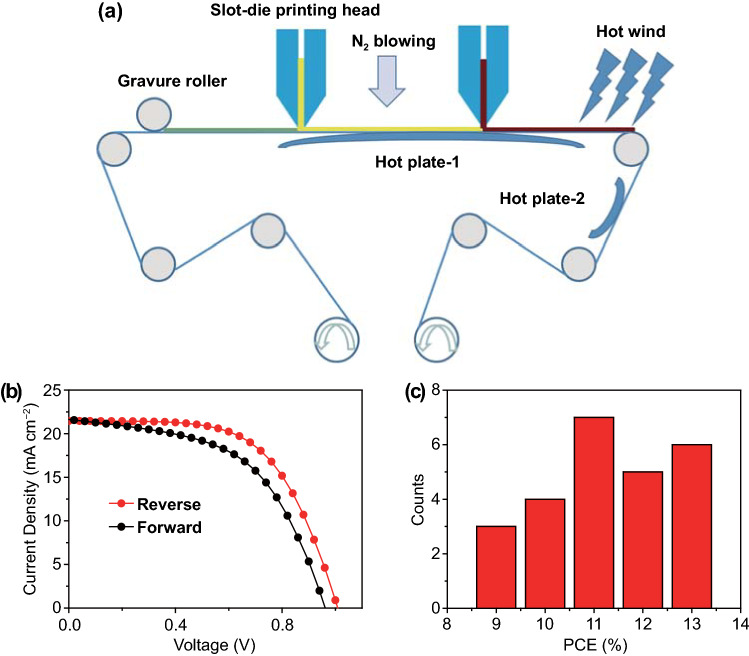
**a** R2R processing set-up for continuous preparation of PSCs. **b** *J–V* curves for the champion device fabricated by R2R coating. **c** The distribution of PCEs obtained from R2R processed 25 devices

The slot-die coating was then transferred to R2R coating process (Fig. 6a). The flexible ITO/PET substrate was continuously moved from an unwind roller, passing the coating head and dryer, and finally to the rewind roller. When each coating run was completed, the substrate was collected on the rewind roller. To start, a thin SnO_2_ film was first coated by micro-gravure printing, followed by slot-die coated of the PbI_2_:CsI layer which was dried by N_2_ blowing, and finally converted to the perovskite film by slot-die coating of FAI/MABr/MACl solution. The detailed R2R coating conditions are described in the experimental session. As shown in Fig. 6a, hot plate-1 is employed to heat the wet film during the coating process as a bed temperature, and the hot plate-2 is used to anneal the dried film. The best performance of PSCs using the R2R coating process present a *V*_*oc*_ of 1.00 V, a *J*_*sc*_ of 21.45 mA cm^−2^, an *FF* of 60.59, and a PCE of 13.00% in a reverse scan (Fig. 6b and Table 2). As shown in Table 2, the hysteresis behavior of R2R coated devices is worse than the slot-die coated ITO/glass substrates. Normally, it is very difficult to control morphology and interface properties of PSCs on flexible substrate using R2R coating process, which would result in more traps in thin film and interface. The trap assisted charge recombination may occur at the interface between the SnO_2_ layer and the perovskite layer. Thus, both ion movement and traps enhance the hysteresis behavior [49–51]. Figure 6c presents the statistical distribution of the PCEs based on 25 PSCs, and the average PCE is about 11.30%.

**Table 2:** The performance parameters of the champion PSCs prepared by slot-die coating were measured under reverse and forward scan directions

Method	Scan direction	*V*_oc_ (V)	*J*_sc_ (mA cm^−2^)	*FF* (%)	*PCE* (%)
Slot-die on glass	Reverse	1.08	22.09	76.01	18.13
Slot-die on glass	Forward	1.08	21.97	75.73	17.97
R2R coating	Reverse	1.00	21.45	60.59	13.00
R2R coating	Forward	0.98	21.56	52.51	11.09

## Conclusions

In summary, high-quality perovskite films are successfully prepared in ambient conditions via slot-die coating on ITO/glass substrates and continuous R2R coating on flexible substrates. The influence of bed temperature on the morphology of PbI_2_:CsI film during the coating was fully investigated. PL and XRD results support the explanation of variable device performances in solar cells when prepared at the different temperatures. Planar n-i-p PSCs with a PCE of 18.13% was achieved by fully slot-die coating. A PCE of 15.10% was achieved for PSCs with an area of 1 cm^2^. Furthermore, the flexible PSC with a PCE of 13.00% was obtained on ITO/PET substrate by the R2R coating process, which is currently one of the highest perovskite performances for fully R2R fabricated PSCs in ambient air. These results will undoubtedly help further pave the way in improving the performance of the large scale PSCs in ambient conditions and catalyse the pursuit towards low cost mass production in the future.

## Supplementary Information

Below is the link to the electronic supplementary material.

Supplementary file1 (PDF 316 kb)
