# Supplementary material for: Fully Roll-to-Roll Processed Efficient Perovskite Solar Cells via Precise Control on the Morphology of PbI2:CsI Layer
Source: Nanomicro Lett. 2022 Mar 25;14:79. doi: 10.1007/s40820-022-00815-7 (PMC8956777; doi:10.1007/s40820-022-00815-7)
Supplement: Supplementary file 1 — Supplementary file1 (PDF 316 kb) [file 40820_2022_815_MOESM1_ESM.pdf]

Supporting Information for

Fully Roll-to-Roll Processed Efficient Perovskite Solar Cells via Precise Control on the Morphology of PbI_2:CsI Layer

Hengyue Li[1, 2], Chuantian Zuo[2], Dechan Angmo[2], Hasitha Weerasinghe[2],

Mei Gao[2, *], Junliang Yang[1, *]

[1] Hunan Key Laboratory of Nanophotonics and Devices, School of Physics and Electronics, Central South University, Changsha, 410083, People's Republic of China

[2] Flexible Electronics Laboratory, CSIRO Manufacturing, Clayton, Victoria 3168, Australia

* Corresponding authors. E-mail: mei.gao@csiro.au (M. Gao), junliang.yang@csu.edu.cn (J.L. Yang)

Supplementary Figures

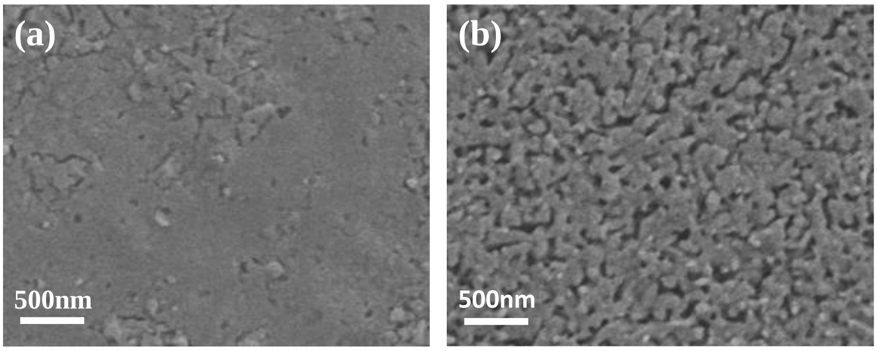

Fig. S1 SEM images of PbI_2:CsI film fabricated by slot-die coating without N_2 blowing (**a**) and with N_2 blowing (**b**)

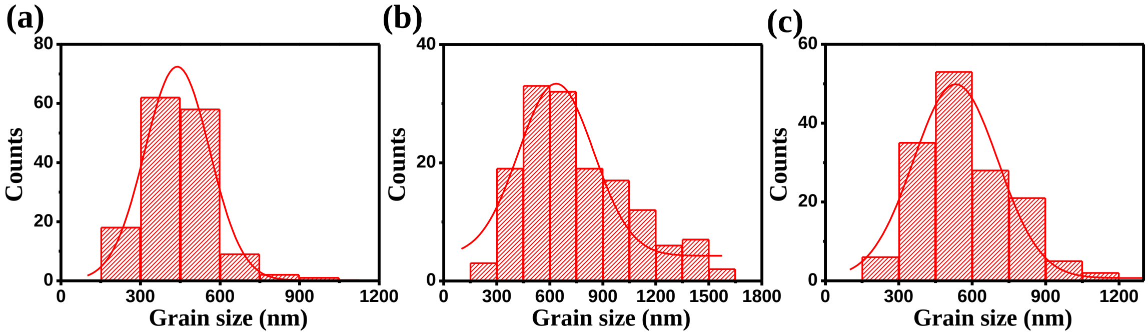

Fig. S2 The grain size distribution of perovskite films prepared on PbI_2:CsI film via slot-die coating at the different substrate temperatures of (**a**) 60℃, (**b**) 70℃, and (**c**) 80℃, respectively

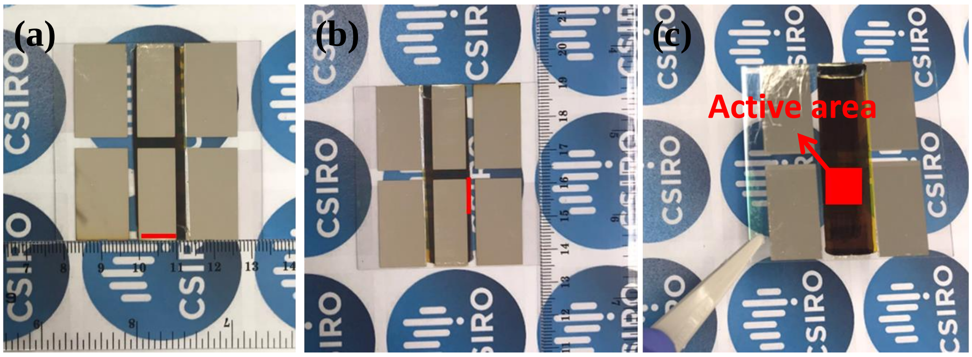

Fig. S3 (**a-c**) Photographs of PSC devices with an area of 1 cm^2
